# The Effectiveness of Dedicated Trauma Operation Theatre and Trauma Intensive Care Unit on the Outcomes of Patients with Traumatic Brain Injury after Emergency Neurosurgery

**DOI:** 10.21315/mjms2022.29.4.12

**Published:** 2021-04-24

**Authors:** Baiduree BORHAN, Wan Mohd Nazaruddin WAN HASSAN, Mohamad Hasyizan HASSAN, Laila AB MUKMIN, Abd Rahman Izaini GHANI

**Affiliations:** 1 Department of Anaesthesiology and Intensive Care, School of Medical Sciences, Universiti Sains Malaysia, Kelantan, Malaysia; 2 Hospital Universiti Sains Malaysia, Universiti Sains Malaysia, Kelantan, Malaysia; 3 Department Neurosciences, School of Medical Sciences, Universiti Sains Malaysia, Kelantan, Malaysia

**Keywords:** traumatic brain injury, operation theatre, Glasgow Coma Scale, neuro-intensive care unit, emergency department

## Abstract

**Background:**

A dedicated trauma operation theatre (TOT) and a trauma intensive care unit (TICU) within the same block as the emergency department (ED) can facilitate immediate management and surgery of patients suffering from traumatic brain injury (TBI). The present study compared the effectiveness and outcomes of TBI management between the TOT-TICU and general OT (GOT) and neuro-ICU (NICU) setups.

**Methods:**

This was a retrospective cohort study involving 120 patients with TBI who were divided into the GOT-NICU (*n* = 63) and TOT-TICU (*n* = 57) groups. Data were obtained from patients’ admission and medical records. Demographic data, durations of specific management phases and outcomes of patients were documented.

**Results:**

In the TOT-TICU group, the duration of transportation from ED to OT [15 (standard deviation [SD] = 15) min versus 45 (SD = 15) min; *P* < 0.001], duration of arrival in OT to incision [50 (SD = 30) versus 70 (SD = 23) min; *P* = 0.005] and duration of transportation from OT to ICU [40 (SD = 17) versus 48 (SD = 30); *P* = 0.005] were significantly shorter than those in the GOT-NICU group. However, the duration of mechanical ventilation, duration of ICU stays, Glasgow Outcome Scale (GOS) upon discharge and GOS at 3-month post-discharge were comparable between both groups.

**Conclusion:**

The TOT-TICU setup shortened the duration of transportation from ED to OT, duration of arrival in OT to incision and duration of transportation from OT to ICU compared with the GOT-NICU setup. Hence, the availability of OT and ICU within the trauma block managed to provide immediate management to TBI patients.

## Introduction

Traumatic brain injury (TBI) is one of the leading causes of death and disability worldwide, with majority of the cases occurring in low-to-middle-income countries (1). According to the Malaysian Registry of Intensive Care Report 2017 (2), brain injury is the second most common cause of intensive care unit (ICU) admission (accounting for 7.1% of all admissions) and the second most common diagnosis leading to in-hospital mortality (accounting for 20.6% of all in-hospital mortalities recorded) in Malaysia.

TBI management encompasses all procedures starting from pre-hospital care and transportation, followed by the first line of treatments in the emergency department (ED) and surgical intervention in the operation theatre (OT), until intensive care unit (ICU) management. Hence, the duration of each management phase may affect the risk of secondary brain insults and outcomes of patients. In previous studies, shortened pre-hospital time significantly reduced the mortality of patients with TBI and the shortening of surgical intervention time increased the survival rate of patients requiring emergency neurosurgical interventions (3–4). In addition, the presence of a dedicated trauma or emergency team and facilities helped reduce the door-to-surgery time (time from ED admission to surgical incision), as the dedicated team could shorten transportation duration and avoid any delays (5–8). Therefore, setting up a dedicated trauma operation theatre (TOT) and trauma ICU (TICU) is anticipated to improve the outcomes of patients suffering from trauma (3, 9).

At our institution, a new trauma block integrating a well-structured ED, TOT and TICU has been functioning since 2015. The TOT is immediately connected to the red zone of the ED and the TICU is located on the first floor of the block. The anaesthesiology team manages the TOT and, the anaesthesiology and ED teams collaboratively manage the five-bed TICU. To the best of our knowledge, this is the only institution in Malaysia where an ICU has been incorporated within the trauma block. This setup is aimed at shortening the time of management (from ED admission to surgery and subsequent TICU admission). Since our hospital is also the main tertiary centre for neurosurgery in East Coast Malaysia, the integrated ED-TOT-TICU setup may help achieve favourable outcomes in patients with TBI. Before the setup of the current trauma block, all emergency surgeries for TBI were conducted in the general OT (GOT), located in a different block and the patients were subsequently admitted to the neuro-ICU (NICU) for further management.

To this end, the present study compared the outcomes [mechanical ventilation duration, ICU stay and Glasgow Outcome Scale (GOS) scores at the time of discharge and 3 months after the discharge] of patients with TBI who underwent emergency neurosurgery and subsequent management in the TOT-TICU or GOT-NICU setup.

## Methods

Patients who were 18 years old–65 years old of age and presented with isolated TBI, regardless of severity, requiring emergency neurosurgical interventions and subsequent management in ICU from 1 June 2015 to 31 June 2019 were included. Patients who were pregnant; who presented with polytrauma, intra-abdominal injury causing dysfunction or requiring surgery, spinal injury or pelvic injury; and who required multiple surgeries (re-craniotomy within 48 h or other surgeries along with neurosurgery) were excluded. Data were collected from the admission records of OT and ICU and medical record of patients.

A total of 120 patients with TBI were included in the study and divided into two groups. The TOT-TICU (*n* = 57) group included patients with TBI who had undergone emergency neurosurgery in the TOT and were subsequently managed in the TICU. The GOT-NICU (*n* = 63) group included patients who had undergone emergency neurosurgery in the GOT and were subsequently managed in the NICU. After reviewing the medical records, demographic data, details of injury and surgery, durations of different management phases from ED to ICU (time of ED admission to transportation to OT, arrival in OT to surgical incision, surgery duration, and time of the completion of surgery to time of arrival in ICU), and patient outcomes (mechanical ventilation duration, ICU stay, and GOS score at discharge and 3 months post-discharge) were recorded.

Sample size was calculated using OpenEpi (http://openepi.com) based on the study of Waqas et al. (10), who assessed the quality of life of patients undergoing decompressive craniectomy (DC) for TBI [*n* = 40; mean (SD) Glasgow Outcome Scale-Extended (GOSE) score, 5.35 (SD = 1.9); mean difference = 1; power of study = 0.8 and significance level *P* = 0.05]. Accordingly, the required sample size was 114, with 57 subjects in each arm. Assuming 10% dropout rate, we included the data of 120 patients in this study.

Data were analysed using SPSS 26.0 (IBM SPSS Inc., NY, USA). Independent *t*-test or Mann-Whitney test was used to compare numerical data and Pearson’s chi-squared or Fisher’s exact test was used to compare categorical data between the two groups. The association amongst numerical variables were determined using Pearson’s correlation analysis and the strength of the association was expressed as follows: fair, 0–0.25; moderate, 0.25–0.5; substantial, 0.5–0.75 and strong, 0.75–1.00. A *P*-value of < 0.05 was considered statistically significant.

## Results

A total of 120 patients were recruited, of which respectively, 63 and 57 were in the GOT-NICU and TOT-TICU groups. Initially, more patients were recruited in the TOT-TICU group, but a few had to drop out (six patients were excluded as they required a second surgery within < 48 h: DC in three patients, laparotomy in one patient and spinal transection in two patients). The demographic data were comparable, with no significant differences between the two groups. Likewise, there were no significant differences between the two groups in terms of injury and type of surgery (Table 1).

Regarding durations of different management phases, the duration of transportation from ED to OT [15 (SD = 15) versus 45 (SD = 15) min; *P* < 0.001], duration of arrival in OT to incision [50 (SD = 30) versus 70 (SD = 23) min; *P* = 0.005] and duration of transportation from OT to ICU [40 (SD = 17) versus 48 (SD = 30); *P* = 0.005] were significantly shorter in the TOT-TICU group than in the GOT-NICU group. However, there were no significant differences in the duration of ED management and surgery between the two groups (Table 2). In addition, there were no significant differences in the outcomes of patients, including the duration of mechanical ventilation, ICU stay, and GOS score at discharge and 3 months post-discharge, between the two groups (Table 3).

Furthermore, the correlations amongst variables that may affect the patients’ GOS score were investigated. The GOS score at discharge was significantly correlated with ED management duration (*r* = −0.189; *P* = 0.039), surgery duration (*r* = −0.182; *p* = 0.046), pre-intubation Glasgow Coma Scale (GCS) score (*r* = 0.578; *P* < 0.001), TBI severity (r = −0.518; *P* < 0.001), mechanical ventilation duration (*r* = −0.362; *P* < 0.001) and ICU stay (*r* = −0.307; *P* < 0.001) in both groups. Furthermore, the 3-month GOS score was significantly correlated with ED management duration (*r* = −0.208; *P* = 0.023), pre-intubation GCS score (*r* = −0.509; *P* < 0.001), TBI severity (*r* = −0.445; *P* < 0.001), mechanical ventilation duration (*r* = −0.428; *P* < 0.001) and ICU stay (*r* = −0.419; *P* < 0.001) in both groups (Tables 4 and 5). There were no significant differences in the correlations of the GOS score with other variables, except those mentioned above between the two groups (Table 5).

## Discussion

The presence of OT and ICU in the same building as ED may improve the quality of management of patients with trauma by shortening the duration from door to definitive surgery until ICU admission. The present study showed that compared with the previous GOT-NICU setup, the new TOT-TICU setup near ED within the same block shortened the duration of transportation from ED to OT, duration of arrival in OT to incision and duration of transportation from OT to ICU. However, the outcomes remained similar between the two setups.

The benefits of dedicated trauma management facilities have been explored in previous studies around the world. This type of management could reduce the morbidity and mortality rates, thereby improving the outcomes of patients with TBI, particularly in a university hospital setting (8). Moreover, a dedicated trauma team of surgeons and anaesthetists who managed trauma cases helped shorten the door-to-surgery time (5), as this team could be notified and readied without delay and the OT was available without interrupting any elective cases. Thus, the surgeon’s delivery of service and job satisfaction could be drastically improved (7). Reduction in duration from arrival to ED to the time of surgery was one of the main goals we sought. In the TOT-TICU setup, the distance from ED to TOT and subsequently to TICU was shorter than that in the GOT-NICU setup. In the GOT-NICU setup, patients had to be transported to the GOT on a stretcher in an ambulance during 2015–2016, when the pathway construction was underway. Subsequently, after the completion of its construction, the patients were transported on a stretcher along the designated pathway (nearly 300 m away). This explains the shorter duration of transportation from ED to OT and possibly of arrival in OT to incision in the TOT-TICU group. Transportation from ED to OT is an important phase in the evaluation of time from injury to definitive management. In patients with isolated severe TBI, shorter duration from injury to surgical intervention significantly reduced the mortality rate (3–4, 6, 11–13). To improve the quality of trauma care in hospitals, a dedicated ICU in the trauma unit should be established; this can significantly reduce the time of transportation from OT to ICU, ultimately improving the outcomes of post-surgical patients with TBI (9). Even though the duration of transportation from ED to OT, duration of arrival in OT to incision and duration of transportation from OT to ICU were significantly shortened in TOT-TICU group, our study did not show any significant differences in outcome parameters such as the duration of mechanical ventilation, duration of ICU stays, GOS upon discharge and GOS at 3-month post-discharge. These might be due to many other confounding factors that might also affect the outcomes, such as the anaesthesia management, neurosurgical procedures, neurosurgical complications, ICU management strategies and complications.

In our analysis of potential factors associated with patient outcomes, the GOS score at discharge was correlated with ED management duration, surgery duration, pre-intubation GCS score, TBI severity, mechanical ventilation duration and ICU stay. Likewise, the 3-month GOS score was correlated with ED management duration, pre-intubation GCS score, TBI severity, mechanical ventilation duration and ICU stay.

The ED team plays an important role in managing patients with TBI, particularly when they are the first to receive the patients and administer medical treatment. Early triage, prompt decision making and treatment administration without delay are key to the successful management of patients with TBI (11). The shorter the duration of ED management, the better the prognosis of patients with TBI due to the lower rate of secondary brain insults. In the present study, this trend was observed in general in both groups. In a multicentre study in Austria (12), the correlations between temporal factors and outcomes were examined; in phase II of that study, some recommendations could avoid time delays (including reduction of duration from admission to OT, first CT scan) and improve the quality of care for patients with TBI and their outcomes [6-month GOSE score].

In this study, the duration of surgery was one of the factors affecting the GOS score of patients with TBI. This may be because the duration of surgery depends on the type and complexity of the surgery as well as the skill and experience of the surgeons (13).

The GCS score is one of the easiest parameters for assessing the severity of TBI in patients presenting to the ED (14). In the present study, the pre-intubation GCS score positively and significantly affected the GOS score at discharge and 3 months post-discharge. Using Pearson’s correlation analysis, we observed that the increased severity of TBI was associated with the reduced GOS score both at discharge and 3 months post-discharge. This result is consistent with some previous reports of the close association between a poor GCS score and poor outcome (15–16). Mechanical ventilation and ICU care are crucial steps in the management of patients with TBI. In the present study, there were no significant differences in the duration of mechanical ventilation and ICU stay between the two groups. However, the durations of mechanical ventilation and ICU stay were negatively correlated with the GOS score at discharge and 3 months post-discharge. Therefore, shorter mechanical ventilation and ICU stay durations may positively affects the GOS score both at discharge and 3 months thereafter. Prolonged ICU stay was associated with an increased rate of nosocomial infections and thus contributed to the mortality of patients in ICU (17).

Furthermore, the correlations of management durations with patient outcomes (mechanical ventilation duration, ICU stay and GOS score at discharge, and 3 months post-discharge) were comparable between the TOT-TICU and GOT-NICU groups (Table 5). This result indicates that the proposed logistics do not actually play a role in improving the outcomes of patients. This finding is similar to the report by Lombardo et al. (9) that the outcomes of patients admitted to the TICU and NICU were similar but better that those of patients admitted to a general ICU (GCS score at discharge and mortality rate in specialised versus general ICU).

The present study has a few limitations. We did not consider the management at the pre-hospital setup in our data collection because of incomplete information. However, we realised the management at the pre-hospital site including the transportation process might affect the outcomes. Only a small number of patients underwent emergency neurosurgery in the TOT because we started performing neurosurgeries at our institution quite recently (since June 2017). Moreover, a limited number of patients were managed in the TOT-TICU setup and majority of the patients who underwent emergency neurosurgery in the TOT were admitted to the NICU or surgical ICU following the surgery in the view of the limited number of beds in the TICU. Hence, a limited number of patients met the inclusion criteria and were recruited in this study. Another limitation is the follow-up duration. Not all patients could be followed-up in our hospital. Therefore, we did not consider their data in this study, as we could not assess their GOS scores. Moreover, some patients defaulted follow-up and were, thus, not considered in this study. Most patients did not follow-up for over 3 months; thus, the GOS or GOSE scores at 6-months and 12-months post-discharge could not be assessed. Finally, as this was a retrospective cohort study, the GOSE score was not assessed as the outcome because patients and their family members need to complete a certain questionnaire to assess the emotional and mental state component of this score, which requires telephone calls and ethical approval.

In future, we would like to suggest a prospective study comparing the similar setup of OT and ICU care of TBI patients with consideration of the involvement of pre-hospital care team, district hospitals that often attending the TBI patients earlier, protocolised OT as well as ICU management and well-planned follow up of the patients.

## Conclusion

The setup of TOT-TICU near ED shortened the duration of transportation from ED to OT, duration of arrival in OT to incision and duration of transportation from OT to ICU compared with the GOT-NICU setup. However, the outcomes were similar between the two setups. Hence, the availability of OT and ICU within the trauma block managed to provide immediate management to TBI patients because the shorter duration of ED management, surgery and transporting to ICU might increase the chance of survival and reduce the potential of secondary insults to the brain.

## Figures and Tables

**Table 1:** Demographic data

Variables	GOT-NICU (*n* = 63)	TOT-TICU (*n* = 57)	*P*-value
Age (years old)	33.4 (14.6)	32.6 (13.4)	0.735
Pre-operative GCS	8.7 (3.3)	7.7 (2.9)	0.068
Severity of TBI			
Mild	10.0 (15.9%)	4.0 (7.0%)	0.083
Moderate	22.0 (34.9%)	14.0 (24.6%)	
Severe	31.0 (49.2%)	39.0 (68.4%)	
Gender			
Male	55.0 (87.3%)	48.0 (84.2%)	0.628
Female	8.0 (12.7%)	9.0 (15.8%)	
ASA physical status classification			
I	50.0 (79.4%)	50.0 (87.7%)	0.364
II	12.0 (19.0%)	7.0 (12.3%)	
III	1.0 (1.6%)	0.0 (0.0%)	
Types of injury			
Extradural haemorrhage	21.0 (33.3%)	14.0 (24.6%)	0.302
Subdural haemorrhage	18.0 (28.6%)	15.0 (26.3%)	
Subarachnoid haemorrhage	3.0 (4.8%)	4.0 (7.0%)	
Contusion	3.0 (4.8%)	0.0 (0.0%)	
Diffuse axonal injury	1.0 (1.6%)	3.0 (5.3%)	
Intraventricular haemorrhage	1.0 (1.6%)	0.0 (0.0%)	
Combination of any two or three injuries	16.0 (25.4%)	21.0 (36.8%)	
Types of surgery			
Craniotomy (+/− EVD)	18.0 (28.6%)	14.0 (24.6%)	0.249
Craniectomy (+/− EVD)	41.0 (65.1%)	34.0 (59.6%)	
Burr hole and EVD insertion	4.0 (6.3%)	9.0 (15.8%)	

Notes: All numerical data are expressed in mean (SD) and categorical data in *n* (%); ASA = the American Society of Anesthesiologists; EVD = external ventricular drain

**Table 2:** Comparison of the duration of different phases of management

Variables	GOT-NICU (*n* = 63)	TOT-TICU (*n* = 57)	*P*-value
ED management duration (min)	204 (170)	235 (118)	0.618^a^
Duration of transportation to OT (min)	45 (15)	15 (15)	^*^< 0.001^a^
Duration of arrival to incision (min)	70 (23)	50 (30)	^*^0.005^a^
Duration of surgery (min)	135 (125)	105 (135)	0.081^a^
Duration of transportation to ICU (min)	48 (30)	40 (17)	^*^0.005^a^

Notes: All numerical data are expressed in median (IqR);

a Mann-Whitney test

**Table 3:** Comparison of the outcome

Variable	GOT-NICU (*n* = 63)	TOT-TICU (*n* = 57)	*P*-value
Duration of mechanical ventilation (days)	3.0 (6.0)	4.0 (5.5)	0.518^a^
Duration of ICU stay (days)	5.0 (7)	5.0 (6)	0.849^a^
GOS upon discharge:			
Death	9.0 (14.3%)	3.0 (5.3%)	0.243^b^
Persistent vegetative stage	1.0 (1.6%)	2.0 (3.5%)	
Severe disability	37.0 (58.7%)	41.0 (71.9%)	
Moderate disability	16.0 (25.4%)	11.0 (19.3%)	
GOS 3-month post-discharge			
Worsening	1.0 (1.6%)	0.0 (0.0%)	0.629^b^
Unchanged	33.0 (52.4%)	31.0 (54.4%)	
Improving	29.0 (46%)	26.0 (45.6%)	

Notes: All numerical data are expressed in in median (IqR) and categorical data in *n* (%);

a Mann-Whitney test;

b Pearson’s chi-squared test

**Table 4:** Correlation between variables and GOS

Case variables	GOS upon discharge		GOS 3-month post-discharge	

	*r*	*P*-value	*r*	*P*-value
ED management duration (min)	−0.189	0.039*	−0.208	0.023*
Duration of transportation to OT (min)	−0.095	0.301	−0.071	0.439
Duration of arrival to incision (min)	−0.060	0.515	0.037	0.688
Duration of surgery (min)	−0.182	0.046*	−0.169	0.065
Duration of transportation to ICU (min)	0.132	0.149	0.057	0.539
Pre-intubation GCS	0.578	< 0.001*	0.509	< 0.001*
Severity of traumatic brain injury	−0.518	< 0.001*	−0.445	< 0.001*
Duration of mechanical ventilation (days)	−0.362	< 0.001*	−0.428	< 0.001*
Duration of ICU stay (days)	−0.307	< 0.001*	−0.419	< 0.001*

Notes: Spearman’s rank correlation coefficient; the strength of the association: Fair: 0–0.25; Moderate: 0.25–0.50; Substantial: 0.50–0.75 and Strong: 0.75–1.00.

* *P*-value < 0.05 indicates statistically significant difference

**Table 5:** Correlation between variables and GOS between GOT-NICU and TOT-TICU

Case variables	GOT-NICU				TOT-NICU			

	GOS during discharge		GOS change 3-month post-discharged		GOS during discharge		GOS change 3-month post-discharged	

	*r*	*P*-value	*r*	*P*-value	*r*	*P*-value	*r*	*P*-value
ED management duration (min)	−0.190	0.135	−0.220	0.083	−0.207	0.123	−0.208	0.121
Duration of transportation to OT (min)	−0.027	0.832	−0.047	0.715	−0.199	0.139	−0.116	0.389
Duration of arrival to incision (min)	−0.063	0.624	0.017	0.892	−0.017	0.898	0.048	0.721
Duration of surgery (min)	−0.197	0.122	−0.075	0.561	−0.174	0.196	−0.281	0.034*
Duration of transportation to ICU (min)	0.121	0.346	0.145	0.258	0.185	0.168	0.002	0.987
Pre-op GCS	0.656	< 0.001*	0.558	< 0.001*	0.496	< 0.001*	0.453	0.000*
Severity of traumatic brain injury	−0.589	< 0.001*	−0.448	< 0.001*	−0.448	< 0.001*	−0.454	0.000*
Duration of mechanical ventilation (days)	−0.424	0.001*	−0.518	< 0.001*	−0.278	0.036*	−0.305	0.021*
Duration of ICU stay (days)	−0.338	0.007*	−0.519	< 0.001*	−0.255	0.056	−0.281	0.034*

Notes: Spearman’s rank correlation coefficient; the strength of the association: Fair: 0–0.25; Moderate: 0.25–0.50; Substantial: 0.50–0.75 and Strong: 0.75–1.00.

* *P*-value <0.05 indicates statistically significant difference
